# Expression analysis of imbalanced genes in prostate carcinoma using tissue microarrays

**DOI:** 10.1038/sj.bjc.6603490

**Published:** 2006-12-05

**Authors:** I Prowatke, F Devens, A Benner, E F Gröne, D Mertens, H-J Gröne, P Lichter, S Joos

**Affiliations:** 1 Division of Molecular Genetics, German Cancer Research Center, Heidelberg, Germany; 2 Division of Biostatistics, German Cancer Research Center, Heidelberg, Germany; 3 Division of Cellular and Molecular Pathology, German Cancer Research Center, Heidelberg, Germany

**Keywords:** prostate cancer, tissue microarray, MYC, *β*-adrenergic receptor kinase (BARK1, GRK2) protein phosphatase (PP1*α*, PP2A)

## Abstract

To identify candidate genes relevant for prostate tumour prognosis and progression, we performed an exhaustive gene search in seven previously described genomic-profiling studies of 161 prostate tumours, and four expression profiling studies of 61 tumours. From the resulting list of candidate genes, six were selected for protein-expression analysis based on the availability of antibodies applicable to paraffinised tissue: fatty acid synthase (FASN), MYC, *β*-adrenergic receptor kinase 1 (BARK1, GRK2) the catalytic subunits of protein phosphatases PP1*α* (PPP1CA) and PP2A (PPP2CB) and metastasis suppressor NM23-H1. These candidates were analysed by immunohistochemistry (IHC) on a tissue microarray containing 651 cores of primary prostate cancer samples and benign prostatic hyperplasias (BPH) from 175 patients. In univariate analysis, expression of PP1*α* (*P*=0.001) was found to strongly correlate with Gleason score. MYC immunostaining negatively correlated with both pT-stage and Gleason score (*P*<0.001 each) in univariate as well as in multivariate analysis. Furthermore, a subgroup of patients with high Gleason scores was characterised by a complete loss of BARK1 protein (*P*=0.023). In conclusion, our study revealed novel molecular markers of potential diagnostic and therapeutic relevance for prostate carcinoma.

Prostate cancer is the most commonly diagnosed noncutaneous neoplasm among males in Western countries and is estimated to result in 28 900 deaths this year in the US alone (Jemal *et al*, 2003). Serum prostate specific antigen (PSA) level and clinico-pathological factors like Gleason score and pT-stage are accepted diagnostic and prognostic factors, which, however, often fail to predict accurately the clinical outcome in individual cases (Frydenberg *et al*, 1997). Therefore, it is of major importance to further elucidate the molecular aberrations present in this disease. This should not only provide a better understanding of the pathomechanisms of this tumour, but also contribute to the development of better diagnostic tools and improved therapeutic strategies.

Array-based screening approaches are widely used to define tumour-specific aberration patterns of the genome and of gene expression. Comparative genomic hybridisation (CGH) and high-resolution array CGH have been successfully applied for the comprehensive screening of whole tumour genomes. In prostate carcinoma, several of these imbalances were shown to correlate with prostate cancer progression. For example, gains of chromosomes 7 and 8q were found to increase with tumour stage and grade (Alers *et al*, 2001). However, owing to the large size of imbalanced chromosomal regions, the group of genes to be considered as possible candidates is usually in the range of several hundreds. Therefore, additional analyses have to be performed in order to identify those genes that represent the most critical candidates relevant for tumour development.

In the present study, we aimed to identify novel relevant biomarkers in prostate carcinoma by combining the results of genomic screening as well as expression profiling data. First, an exhaustive search for imbalanced chromosomal regions from seven CGH and array CGH studies was performed (Sattler *et al*, 1999; Alers *et al*, 2001; Verdorfer *et al*, 2001; Zitzelsberger *et al*, 2001; Steiner *et al*, 2002; Wolter *et al*, 2002; Paris *et al*, 2003). Next, the results were compared to a recent meta-analysis of four independent expression array data sets (Rhodes *et al*, 2002) in order to define candidate genes located within regions recurrently changed in copy number. Of the candidate genes identified, six were selected for protein-analysis based on the availability of antibodies suitable for paraffin-embedded tissue. Detailed immunohistochemical analysis was performed in a cohort of prostate carcinomas using tissue microarray technology. The correlation of protein expression with Gleason score and pT-stage indicated new candidates involved in the pathomechanisms of prostate cancer and potential diagnostic markers.

## MATERIALS AND METHODS

### Patient material

Formalin-fixed, paraffin-embedded primary prostate adenocarcinomas resected between 1998 and 2003 were diagnosed in the Division of cellular and molecular pathology at the German Cancer Research Centre, Heidelberg. All samples were taken from a tumour bank, and approval to link laboratory data to clinical data was obtained from the Institutional review board. This procedure is compliant with the official statement of the National ethics council of the Federal Government of Germany (March 2004). The study included 175 patients. After construction of the tissue microarray, every 30^th^ tissue microarray slide was evaluated by a pathologist (H-J G). Both tumour and adjacent benign prostatic hyperplasia (BPH) material could be analysed in 82 cases. In 49 cases, only carcinoma and in 44 cases only BPH could be analysed. The median age at the time of diagnosis was 67 years (range 47–89). Clinico-pathological data including Gleason score and pathological staging according to the tumour node metastasis classification are summarised in Table 1. For univariate statistical analysis, Gleason scores were categorised as Gs5–6, Gs7, and Gs8–9 and pT-stage was categorised as pT2 *vs* pT3–4. For multivariate statistical analysis, clinico-pathological criteria were categorised into two groups each: well to moderately differentiated tumours with Gleason scores 2–6 indicating a good prognosis were compared with poorly differentiated tumours with Gleason scores 7–9 indicating more aggressive tumours and a worse prognosis; pT-stage was categorised as organ-confined (pT2) *vs* extraprostatic (pT3–pT4).

### Analysis of genomic and expression data sets

To reveal the most abundant chromosomal imbalances detected in prostate carcinoma, we performed an exhaustive search of six CGH studies, including a total of 145 primary prostate cancers (Sattler *et al*, 1999; Alers *et al*, 2001; Verdorfer *et al*, 2001; Zitzelsberger *et al*, 2001; Steiner *et al*, 2002; Wolter *et al*, 2002; Paris *et al*, 2003) as well as one array CGH study of 16 primary prostate cancers (Paris *et al*, 2003). In order to identify candidate genes that may be directly affected by these chromosomal imbalances, we screened a list of the top 250 most up-regulated and down-regulated genes as revealed by a recent meta-analysis of four independent expression microarrays including 61 prostate cancer samples (Rhodes *et al*, 2002).

### Construction of a prostate carcinoma tissue microarray

Tissue microarrays were constructed as originally described (Kononen *et al*, 1998). Briefly, representative tumour and benign hyperplastic regions were marked by a pathologist (H-JG) on haematoxylin and eosin (H&E) stained sections from each paraffinised tumour block. Tissue cylinders with a diameter of 0.6 mm were punched from selected areas of each ‘donor’ block and transferred to a recipient paraffin block using a tissue chip microarrayer (Beecher Instruments, Silver Spring, MD, USA). Two to three tissue cores per tumour or BPH sample were isolated and arrayed, summing up to a total of 651 tissue cores. According to previous studies (Nocito *et al*, 2001; Torhorst *et al*, 2001), this is sufficient to compensate for the heterogeneous composition of prostate carcinomas in tissue microarray analyses.

For immunohistochemistry (IHC) analyses, 5 *μ*m sections from the recipient block were prepared. Sections from the front, the middle and the rear part of the block were H&E stained and reviewed again by a pathologist (H-JG) in order to distinguish benign from malignant tissue.

### Immunohistochemistry

Immunohistochemistry was performed using an avidin–biotin complex (ABC) approach (Vectastain ABC Kit, Vector Laboratories Inc., Burlingame, CA, USA). Sections were deparaffinised and epitopes were retrieved by heating in a microwave oven with 10 mM citrate buffer (pH 6.0) (for MYC: 10 mM Tris, 1 mM ethylenediaminetetraacetic acid (EDTA), (pH 9.0) for 20 min. Endogenous peroxidase activity was inhibited by 3% hydrogen peroxide. Unspecific binding was blocked with 1% normal serum (for NM23-H1: 3% bovine serum albumin (BSA). Biotinylated anti-rabbit, anti-mouse, anti-goat, and anti-sheep immunoglobulins (Vector Laboratories Inc., Burlingame, CA, USA) were used as secondary antibodies. For colour development, the specimens were incubated with 3,3′-diaminobenzidine hydrochloride (DAB) supplemented with hydrogen peroxide (DAB Substrat kit, Linaris, Wertheim-Bettingen, Germany) and afterwards counterstained in haematoxylin. Primary anti-human antibodies were anti-NCL-cMYC (9E11), mouse monoclonal diluted 1 : 175 (Novocastra Laboratories Ltd, Newcastle, UK), anti-fatty acid synthase (FASN) rabbit polyclonal diluted 1 : 150 (Assay Design Inc., Ann Arbor, MI, USA), anti-BARK1 (5D5) mouse monoclonal diluted 1 : 300 (Invitrogen (Zymed), Paisley, UK), anti-NM23-H1 (NM301) mouse monoclonal diluted 1 : 50, anti-PP1*α* (N-19) goat polyclonal diluted 1 : 150 and anti-PP2A (FL-309) rabbit polyclonal diluted 1 : 100 (Santa Cruz Biotechnology, Inc., CA, USA). Anti-PP2A does not distinguish between the highly homologous (97%) *α*- and *β*-isoforms of PP2A. Immunohistochemistry was performed on consecutive sections. One section was immunostained with anti-CK5 sheep polyclonal antibody (1 : 4000, The Binding Site, Birmingham,UK) for cytokeratin 5, a specific marker for basal cells, to distinguish benign from malignant glands in each tissue core. Glandular tissue that had shown positive results in former experiments served as positive control. Negative controls were prepared by omitting the primary antibody.

### Scoring of antibody staining

Cytoplasmic (MYC, BARK1, PP1*α*, PP2A, FASN) and nuclear immunoreactivity (PP1*α*, PP2A, NM23-H1) were scored separately according to staining intensity and graded semi-quantitatively as negative (−), weakly positive (+), moderately positive (++), and strongly positive (+++). For statistical analyses, the immunostaining classifications were reduced to two categories: negative and positive. The negative category included weakly positive staining (−, +) owing to the low numbers of completely negatively stained samples. Each antibody was evaluated in a double-blind fashion. The mean of all scores per specimen was used as a single value for statistical analysis.

### Statistical analyses

Statistical analyses were performed using the statistical software package SPSS 12.0 (SPSS GmbH Software, Munich, Germany). Univariate analyses between immunohistochemical staining and clinico-pathological variables were performed using *χ*^2^ and Fisher's exact tests. The familywise error rate was adjusted for 5% false positives using Bonferroni–Holm correction from the software package R, version 2.1.0. These error rates were independently calculated for the clinico-pathological parameters Gleason score and pT-stage. Yet, *P*-values mentioned in the text below refer to the unadjusted *P*-values unless stated otherwise. For multivariate analysis, binary logistic regression analysis together with a moderate backward selection was applied to all patients, from whom complete clinico-pathological data sets as well as IHC results were available (significance level for staying in the model: 0.5). *P*-values ⩽0.05 were considered as statistically significant in all analyses.

## RESULTS

### Selection of candidate genes by correlation of genomic and expression data sets

To establish a list of candidate genes relevant for prostate carcinogenesis, we first performed an exhaustive search of six previously published CGH analyses (145 primary prostate cancers) and one array CGH analysis (16 primary prostate cancers) (see Material and Methods). Copy number gains most frequently affected regions on the chromosomal arms 17p, 8q, 7q, 3q, 7p, 17q, 1p, 19q, 20q, 16p, 11q, 12q, 19p, and 9q in declining frequency (21 to 8%), whereas copy number losses particularly involved subregions on 8p, 13q, 6q, 16q, 18q, 5q, 2q, and 4q in 28–9% of the cases. In order to narrow down the range of possible candidate genes from these regions, only the top 250 upregulated and the top 250 downregulated genes in prostate carcinoma (Rhodes *et al*, 2002) were further analysed in this study. We found 32 genes located on frequently over represented bands and 28 genes on frequently underrepresented bands (Supplementary Tables 2.1 and 2.2). These included MYC (8q24), BARK1 (11q13), metastasis suppressor NM23-H1 (17q21), FASN (17q25) the catalytic subunit of protein phosphatase PP1*α* encoded by *PPP1CA* (11q13) as well as protein phosphatase PP2A (*ß*-isotype) encoded by *PPP2CB* (8p11–p12). These genes were further analysed in detail in a collection of prostate carcinomas using antibodies, which had been established for immunhistochemical detection against the corresponding proteins in paraffin-embedded tissue material. The six candidate genes, their respective chromosomal localisation as well as the frequency and rank of genomic and expression alterations, respectively, are summarised in Table 2.

### Subcellular expression pattern of candidate proteins

**Figure 1:**
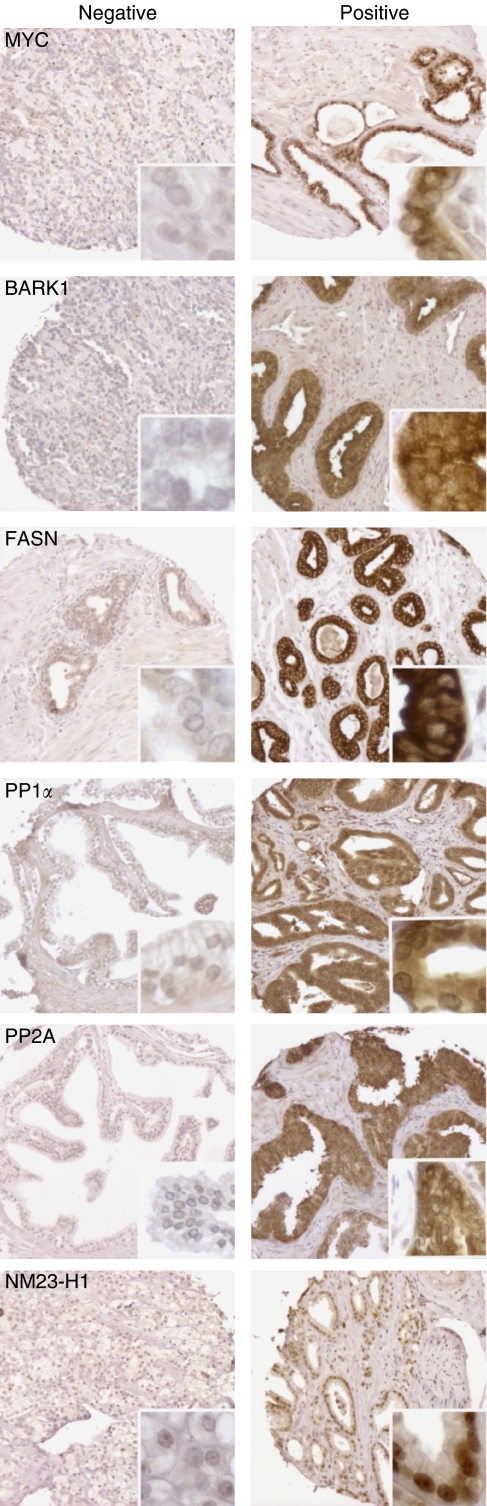
Examples of immunohistochemical staining of MYC, BARK1, FASN, PP1*α*, PP2A, and NM23-H1 on whole-tissue microarray cores (0.6 mm in diameter) and subcellular staining pattern (inserts). Negative immunostaining: left column, positive immunostaining: right column. The category of negative staining included weakly positive staining.

Examples of typical staining patterns from the antibodies used are shown in Figure 1. Fatty acid synthase immunostaining was homogeneously dispersed throughout the cytoplasm as described before (Swinnen *et al*, 2002; Rossi *et al*, 2003). Subcellular staining of MYC was predominantly cytoplasmic: in BPH, perinuclear focal granular immunostaining was observed on the luminal site of the cells, whereas malignant secretory cells showed a diffuse cytoplasmic immunostaining. This subcellular staining pattern is in accordance with previous observations using the same antibody in prostate cancer tissue (Jenkins *et al*, 1997). Beta-adrenergic receptor kinase 1 immunostaining was observed in the cytoplasm of glandular cells. Concerning PP1*α* and PP2A, a cytoplasmic as well as a nuclear staining was observed. Cytoplasmic and nuclear localisation of nm23-H1 were observed as recently shown in IHC of the same antibody (Forus *et al*, 2001).

### Correlation of protein expression and clinico-pathological parameters using a prostate cancer tissue microarray

#### Univariate analyses

In order to test whether the expression of the selected proteins correlates with clinico-pathological parameters, we constructed a tissue microarray. The tissue microarray contained 651 analysable tissue cores from 131 different tumour samples and 126 BPH from 175 prostate cancer patients. Univariate analyses revealed a significant difference between tumour and benign hyperplastic tissue for immunohistochemical staining results of FASN (*P*<0.001), MYC (*P*<0.001), and PP1*α* (*P*=0.020), indicating a role of these proteins in tumour initiation (Table 3).

**Figure 2:**
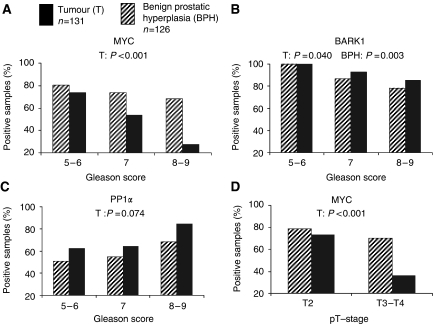
Correlation of clinico-pathological parameters with immunostaining of MYC, BARK1 and PP1*α* in prostate tumour and BPH. MYC immunostaining negatively correlated with Gleason score (**A**), and pT-stage (**D**) in tumour samples. Negative BARK1 immunostaining characterised a subgroup of patients with high Gleason scores (**B**). Protein phosphatase1*α* immunostaining positively correlated with Gleason score (**C**) (Gs8–9 *vs* 5–7: *P*=0.001). *P*-values are derived from *χ*^2^test or Fisher's exact test.

Univariate correlations of protein expression with Gleason score and pT-stage were found for MYC, BARK1 and PP1*α* (Table 4, Figure 2), indicating a role of these proteins in tumour progression. MYC immunostaining strongly decreased from low to high Gleason scores as well as pT-stages (*P*<0.001 each). Beta-adrenergic receptor kinase 1 immunostaining decreased from low to high Gleason scores (*P*=0.040). A negative correlation of BARK1 immunostaining with Gleason score was also stated for the benign hyperplastic tissue areas of the patients (*P*=0.003). Protein phosphatases PP1*α* immunostaining was constant in low and intermediate Gleason scores (Gleason scores 5–7), but increased in high Gleason scores (*P*=0.001). For PP2A, FASN, and NM23-H1, no correlations with the prognostic clinico-pathological parameters were stated in univariate analysis (Table 4). None of the proteins analysed correlated with patient's age (data not shown).

#### Multivariate analyses

Logistic regression was used to predict the outcome variables Gleason score (Gs2–6 *vs* Gs7–9) and pT-stage (organ-confined *vs* extraprostatic) (Table 5). All proteins in this study were included in the model. A moderate backward selection was applied in order to exclude factors that were not relevant. MYC, BARK1, and FASN protein expression were statistically significant predictors of Gleason score (*P*<0.001, *P*=0.023, *P*=0.036), representing tumour differentiation. High odds ratios (OR) and 95% confidence intervals (CI) of MYC (OR=7.143 [2.079–24.390]) and FASN (OR=3.106 [1.035–9.259]) further support their significant correlation with Gleason score. For BARK1, odds ratio and 95% CI were not computable because negative BARK1 immunostaining was exclusively found in patients with high Gleason scores and never in cases with low Gleason scores. Finally, MYC protein expression was the only factor predicting pT-stage (*P*<0.001, OR=6.993 (2.387–20.408)), that is in agreement with the role of MYC in growth control.

In summary, logistic regression analysis identified MYC, BARK1, and FASN protein expression predicting Gleason score. MYC protein expression additionally predicted pT-stage.

## DISCUSSION

The aim of this study was to identify proteins that might be of potential biological and clinical relevance in the field of prostate cancer. Six candidates, from whom antibodies for IHC analyses are available, were analysed in detail in a large tumour collection using tissue microarray technology: FASN, BARK1, PP1*α*, PP2A, NM23-H1, and MYC.

Fatty acid synthase is an androgen-regulated enzyme required for *de-novo* lipogenesis. Fatty acid synthase mRNA and protein upregulation is one of the earliest and most common events in the development of prostate carcinoma, and a strong association between FASN and tumour initiation has been shown (Swinnen *et al*, 2002; Rossi *et al*, 2003). Accordingly, we found FASN expression to be much stronger in tumours than in BPH. Further, FASN expression correlates with high Gleason scores in multivariate analysis, which is in accordance with previous reports (Shurbaji *et al*, 1996). These results demonstrate the validity of our experimental approach and further underline the role of FASN as a molecular marker and therapeutic target in prostate cancer as proposed previously (Baron *et al*, 2004).

*β*-adrenergic receptor kinase , the second candidate defined in this study, has not been examined in the context of prostate cancer progression before. *β*-adrenergic receptor kinase specifically desensitises agonist-occupied *β*-adrenergic receptors (Pierce *et al*, 2002). Enhanced adrenergic receptor signalling has been shown to be involved in the development of androgen-independent prostate cancer cell proliferation (Kasbohm *et al*, 2005). We found tumours negative for BARK1 exclusively in cases with high Gleason scores (Gs7–9). According to the function of BARK1, a highly sensitise adrenergic receptors and enhanced signalling would be expected under these conditions. It would be of interest to test whether downregulation of BARK1 may constitute a mechanism to trigger androgen-independent tumour growth. Furthermore, negative BARK1 expression may indicate patients for which an androgen-ablation therapy may be futile.

Two of the novel candidates analysed represent protein phosphatases, PP1*α* and PP2A. Both are involved in signal transduction, apoptosis, protein synthesis and intracellular transport, RNA splicing, and cell-cycle regulation (Janssens and Goris, 2001; Garcia *et al*, 2003). The catalytic subunit of PP1*α* is located on chromosomal band 11q13 that showed frequent gain in array CGH analysis (Paris *et al*, 2003). Accordingly, we found enhanced cytoplasmic PP1*α* immunostaining in tumours *vs* BPH. Further, enhanced cytoplasmic PP1*α* immunostaining correlated with high Gleason scores (8–9), suggesting a role of this gene in tumour progression.

Owing to their role as regulators of the cell cycle as well as in apoptosis, protein phosphatases are increasingly discussed as candidates for therapeutic interference (Berndt *et al*, 1997; McCluskey *et al*, 2002). Inhibitors of protein phosphatases exert anticancer activity and have already been used in the treatment of primary hepatoma and upper gastrointestinal carcinoma (Berndt *et al*, 1997; McCluskey *et al*, 2002). According to the results presented, such inhibitors might be applicable in future therapeutic strategies to prostate cancer after considering the PP1*α* expression status. In contrast to PP1*α*, PPP2CB, a catalytic subunit of PP2A, did not correlate with the clinico-pathological factors analysed suggesting that PP2A does not play a prominent role in prostate cancer.

Another candidate analysed in our study is the metastasis suppressor NM23-H1, which is involved in the synthesis of deoxynucleotides (Steeg, 2003). In prostate cancer, an inverse relationship between NM23-H1 expression and metastatic status was described, but a correlation with the progression of primary tumours is controversially discussed (Konishi *et al*, 1993; Igawa *et al*, 1994). According to the present study, no correlation with clinico-pathological parameters of primary prostate carcinomas was found.

In contrast, the sixth candidate, MYC, was not only found to be differentially expressed in tumour *vs* BPH, but also correlated with both Gleason score and pT-stage. Interestingly, MYC immunostaining decreased with tumour progression.

The c-myc gene is located on chromosome 8q, the gain of which is an early event in prostate carcinogenesis (Alers *et al*, 2001). Amplification of the c-myc locus 8q24 is linked to higher Gleason scores (Qian *et al*, 2002) and a poor prognosis (Sato *et al*, 1999; Tsuchiya *et al*, 2002). Concerning MYC protein expression, only two studies have been published for prostate cancer tissue so far. Royuela *et al* (2000) described MYC immunostaining to be positive in prostate tumours and BPH (Jenkins *et al*, 1997; Royuela *et al*, 2000), which is in concordance with our observations. The second study indicated that cytoplasmic immunostaining of MYC increased with copy number gains of chromosome 8, which in turn correlated with the occurrence of lymph node metastases (Jenkins *et al*, 1997). Although a copy number gain would also be expected for the tumours analysed in the present study, we found a negative correlation of MYC immunostaining and tumour progression.

Our data indicate that MYC protein expression is not primarily dependent on chromosome 8 or c-myc gene copy number. This is supported by various findings described in previous studies. Thus, although a normal chromosomal complement can be expected in BPH, like in normal prostate, MYC immunostaining was described to be negative in normal prostate from young men but positive in BPH (Royuela *et al*, 2000). Further, in prostate cancer cell lines, MYC transcript levels were similar despite the fact that the c-myc gene was amplified in some cell lines but not in others (Tsuchiya *et al*, 2002). Finally, in human hepatocellular carcinoma, a similar discrepancy between c-myc gene amplification and immunostaining was reported as in the present study: despite a c-myc amplification, human hepatocellular carcinoma had less nuclear and cytoplasmic MYC imunostaining than noncancerous livers without c-myc amplification (Chan *et al*, 2004).

These findings implicate that MYC expression in prostate cancer is not simply regulated by a gene dosage effect. We hypothesise that MYC overexpression may be favourable for the proliferation of benign hyperplastic cells, but may not be required for the maintenance and progression of the tumour. It should also be noted that MYC is not only involved in proliferation, but also in apoptotic pathways (Chan *et al*, 2004). Therefore, if MYC is downregulated during tumour progression as was observed in the present study, this might reflect a mechanism to inhibit MYC-activated apoptosis.

In conclusion, we showed that MYC immunostaining predicts Gleason score and furthermore pT-stage, which represents the two clinico-pathological parameters that correlate best with prostate cancer-specific survival. However, the precise role of MYC in prostate cancer progression needs further clarification.

The combined genomic and expression profiling analysis described in this study suggested an upregulation of FASN, BARK1, MYC, PP1*α*, and NM23-H1 expression and a downregulation of PP2A expression in prostate tumours. Indeed, such a relation was observed for FASN and PP1*α*, indicating a gene dosage effect, but not for the other candidate proteins examined. In fact, multiple mechanisms contribute to the development and progression of prostate cancer. For example, genomic copy number changes may initially result in an altered protein expression, but this effect might be replaced by other mechanisms during later phases of tumour development.

In conclusion, we demonstrated a novel approach providing candidate genes of potential clinical and biological relevance. Immunohistochemical analyses revealed several candidates correlating with clinico-pathological factors that are markers for prostate cancer-specific survival. The impact of the candidates on prostate carcinogenesis and their potential clinical application remain to be analysed in further detailed studies.

## Figures and Tables

**Table 1:** Clinico-pathological features of 175 patientsa,^b^

**Clinico-pathological factor**	**Classification**	**Tumour (no. of cases^c^)**	**BPH^d^ (no. of cases^c^)**
Gleason score	2	2	2
	4	2	3
	5	15	25
	6	30	34
	7	31	24
	8	36	21
	9	9	6
Subtotal		125	115
			
pT-stage	pT2	55	84
	pT3	44	31
	pT4	5	2
Subtotal		109	113
Total		131	126

BPH=benign prostatic hyperplasias.

a Only cases evaluated are listed above. Both tumour material and BPH material could be analysed from 82 patients. Additionally, carcinoma was analysable in 49 cases and BPH in another 44 cases.

b Age range 47–89 years, median 67, mean 66.

c Clinico-pathological data were not available for all patients.

d Benign prostatic hyperplasias adjacent to tumours with the denoted stage or Gleason score.

**Table 2:** Candidates for IHC analysis, derived by correlation of expression array data with CGH and array CGH data

**Gene^a^**	**Gene name^a^; alias**	**Chromosomal location**	**CGH^b^ (*n*=145), gains**	**Array CGH^c^ (*n*=16), gains**	**Expression array meta-analysis^d^ (*n*=61), rank no.^e^**
*NME1*	*Non-metastatic cells 1, protein (NM23a) expressed in*	17q21.3	15.2%	2/16	1
*FASN*	*Fatty acid synthase; FAS, OA-519*	17q25	20.7%	3/16	6
*MYC*	*v-myc myelocytomatosis viral oncogene homolog (avian)*	8q24.12–q24.13	15.2%	0/16	46
*ADRBK1*	*Adrenergic, beta, receptor kinase 1; GRK2; BARK1*	11q13	8.3%	3/16	131
*PPP1CA*	*Protein phosphatase 1, catalytic subunit, alpha isoform*	11q13	8.3%	3/16	236
					
			**Deletions**	**Deletions**	
*PPP2CB*	*Protein phosphatase 2 (formerly 2A), catalytic subunit, beta isoform*	8p12–p11.2	23.5%	5/16	66

CGH=comparative genomic hybridisation; IHC=immunohistochemistry.

a HUGO approved gene symbol and name.

b Alers *et al* (2001), Sattler *et al* (1999), Steiner *et al* (2002), Verdorfer *et al* (2001), Wolter *et al* (2002) and Zitzelsberger *et al* (2001).

c Paris *et al* (2003).

d Rhodes *et al* (2002).

e Rank in lists of the 500 most upregulated and downregulated genes in prostate cancer *vs* benign prostate tissue.

**Table 3:** Immunostaining of all analysed proteins in BPH and prostate tumour samples

	**IHC positive staining (%) (*n* positive/*n* total)**					
**No. of cases (*n*)**	**MYC^a^**	**BARK1^a^**	**PP1*α*^a^**	**FASN^a^**	**NM23-H1^b^**	**PP2Aa,^b^**
Tumour (*n*=131)	52%	93%	71%	54%	59%	54%
BPH (*n*=126)	78%	92%	56%	15%	61%	56%
						
*P*-value	<0.001	0.807	0.020	<0.001	0.685	0.891
Adjusted *P-*value^c^	<0.001	1.000	0.120	<0.001	1.000	1.000

BARK1**=***β*-adrenergic receptor kinase 1; FASN**=**Fatty acid synthase; PP2A=protein phosphatases.

a Cytoplasmic immunostaining.

b Nuclear immunostaining.

c In order to correct for multiple testing *P*-values were adjusted using Bonferroni–Holm correction.

**Table 4:** Correlation between clinico-pathological factors and positive immunostaining of all proteins analysed

			**Positive IHC staining (%) (*n* positive/*n* total)**											
	**No. of cases**		**MYC^a^**		**BARK1^a^**		**PP1*α*^a^**		**FASN^a^**		**NM23-H1^b^**		**PP2Aa,^b^**	
**Clinico-pathological factor**	**Tumour (*n*=131)**	**BPH (*n*=126)**	**Tumour**	**BPH**	**Tumour**	**BPH**	**Tumour**	**BPH**	**Tumour**	**BPH**	**Tumour**	**BPH**	**Tumour**	**BPH**
*Gleason score*														
5–6	45	59	74%	80%	100%	100%	63%	51%	54%	16%	59%	55%	60%	60%
7	31	24	54%	74%	93%	87%	64%	55%	59%	18%	62%	71%	60%	61%
8–9	45	27	28%	68%	85%	78%	84%	68%	57%	14%	59%	76%	43%	50%
	121^c^	110^c^												
*P*-value			<0.001	0.501	0.040	0.003	0.074^d^	0.385	0.910	0.918	0.974	0.134	0.280	0.686
Adjusted *P*-value^e^			0.002	1.000	0.240	0.024	0.052	1.000	1.000	1.000	1.000	0.804	1.000	1.000
														
*pT-stage*														
pT2	60	80	73%	79%	96%	92%	66%	58%	59%	14%	61%	60%	55%	54%
pT3-pT4	49	33	36%	70%	93%	87%	73%	48%	63%	19%	60%	70%	55%	53%
	109^c^	113^c^												
*P*-value			<0.001	0.324	0.657	0.477	0.515	0.392	0.832	0.556	1.000	0.375	1.000	1.000
Adjusted *P*-value^e^			0.006	1.000	1.000	1.000	1.000	1.000	1.000	1.000	1.000	1.000	1.000	1.000

BARK1**=***β*-adrenergic receptor kinase 1; FASN**=**Fatty acid synthase; PP2A=protein phosphatases.

a Cytoplasmic immunostaining.

b Nuclear immunostaining.

c No. of samples for which the respective clinico-pathological data were available. Not all BPH samples correspond to the same patients as the tumour samples.

d For Gleason scores 5–7 *vs* 8–9, *p*=0.001.

e *P*-values of Gleason score and pT-stage were corrected for multiple testing using Bonferroni-Holm for all proteins in this study.

**Table 5:** Multivariate analyses for the prediction of clinico-pathological parameters by immunostaining results of MYC, BARK1, PP1*α*, PP2A, NM23-H1 and FASN

**Clinico-pathological factor**	**Cases in analysis**	**Results^a^**	***P*-value^b^**	**OR (95% CI)**
Gleason score (Gs2-6/Gs7-9)	82	MYC	<0.001	7.143 (2.079–24.390)
		BARK1	0.023	—c
		FASN	0.036	3.106 (1.035–9.259)
				
pT-stage (pT2/pT3-pT4)	77	MYC	<0.001	6.993 (2.387–20.408)

BARK1**=***β*-adrenergic receptor kinase 1; FASN=Fatty acid synthase; PP2A=protein phosphatases.

a Results of logistic regression analysis with a moderate backward selection.

b *P*-value of likelyhood-ratio test.

c Numerically not computable as each of the eight negative BARK1 staining results was exclusively found in advanced Gleason scores (Gs7–9) (see Table 4).
