# Supplementary material for: Expression analysis of imbalanced genes in prostate carcinoma using tissue microarrays
Source: Br J Cancer. 2006 Dec 5;96(1):82–8. doi: 10.1038/sj.bjc.6603490 (PMC2360197; doi:10.1038/sj.bjc.6603490)
Supplement: Supplementary Table 2.1 [file 6603490x1.doc]

| **Supplementary Table 2.1** Up-regulated genes (from top 250) on chromosomal bands with frequent (> 5%) gains | | | | |  |
| --- | --- | --- | --- | --- | --- |
|  |  |  |  |  |  |
|  |  |  |  |  |  |
|  |  |  | **CGH*** | **Array CGH**** | **Expression array meta-analysis***** |
|  |  | **Chromosomal** | n=145 | n=16 | n=61 |
| **Gene** # | **Gene Name** #**; Alias** | **Location** | **Gains** | **Gains** | **Rank No.** § |
|  |  |  |  |  |  |
| *ARMET* | *arginine-rich, mutated in early stage tumours; ARP* | 3q21.1 | 7.6% | 2/16 | 2 |
| *PDIA5* | *protein disulfide isomerase-associated 5; PDIR* | 3q21.1 | 7.6% | 2/16 | 7 |
| *ATP2C1* | *ATPase, Ca++ transporting, type 2C, member 1* | 3q21.3 | 7.6% | 2/16 | 245 |
| *MRPL3* | *mitochondrial ribosomal protein L3; RPML3* | 3q21-q23 | 8.3% | 2/16 | 123 |
| *EIF3S9* | *eukaryotic translation initiation factor 3, subunit 9 eta, 116kDa* | 7p22.3 | 8.9% | 1/16 | 242 |
| *ABHD11* | *abhydrolase domain containing 11* | 7q11.23 | 11.7% | 1/16 | 197 |
| *TBL2* | *transducin (beta)-like 2, WBSCR13; WS-betaTRP* | 7q11.23 | 11.7% | 1/16 | 233 |
| *STEAP1* | *six transmembrane epithelial antigen of the prostate 1* | 7q21 | 13.8% | 1/16 | 150 |
| *GUSB* | *glucuronidase beta* | 7q21.11 | 13.8% | 1/16 | 116 |
| *SND1* | *staphylococcal nuclease domain containing 1; p100* | 7q31.3 | 12.4% | 2/16 | 69 |
| *IMPDH1* | *IMP (inosine monophosphate) dehydrogenase 1* | 7q31.3-q32 | 13.1% | 2/16 | 144 |
| *RAB2* | *member RAS oncogene family* | 8q12.1 | 10.3% | 0/16 | 106 |
| *TPD52* | *tumour protein D52, N8L; hD52* | 8q21 | 19.9% | 0/16 | 20 |
| *PVT1* | *Pvt-1 oncogene homolog, MYC activator (mouse)* | 8q24 | 15.2% | 0/16 | 250 |
| *MYC* | *v-myc myelocytomatosis viral oncogene homolog (avian)* | 8q24.12-q24.13 | 15.2% | 0/16 | 44 |
| *TOR1B* | *torsin family 1, member B (torsin B)* | 9q34 | 6.2% | 1/16 | 241 |
| *KIAA0310* | *KIAA0310* | 9q34.3 | 6.2% | 1/16 | 199 |
| *ADRBK1* | *adrenergic, beta, receptor kinase 1; GRK2; BARK1* | 11q13 | 6.9% | 3/16 | 131 |
| *PPP1CA* | *protein phosphatase 1, catalytic subunit, alpha isoform* | 11q13 | 6.9% | 3/16 | 236 |
| *LIG3* | *ligase III, DNA, ATP-dependent* | 17q11.2-q12 | 15.9% | 3/16 | 17 |
| *MAP2K3* | *mitogen-activated protein kinase kinase 3; MEK3; MKK3* | 17q11.2 | 15.9% | 3/16 | 212 |
| *ACACA* | *acetyl-Coenzyme A carboxylase alpha* | 17q21 | 15.2% | 2/16 | 196 |
| *JUP* | *junction plakoglobin* | 17q21 | 15.2% | 2/16 | 204 |
| *NME1* | *non-metastatic cells 1, protein (NM23a) expressed in; NM23-H1* | 17q21.3 | 15.2% | 2/16 | 4 |
| *SLC35B1* | *solute carrier family 35, member B1; UGTREL1* | 17q21-q32 | 19.3% | 2/16 | 54 |
| *FASN* | *fatty acid synthase; FAS, OA-519* | 17q25 | 18.6% | 18.6% | 6 |
| *STRA13* | *stimulated by retinoic acid 13* | 17q25.3 | 18.6% | 18.6% | 30 |
| *ARHGDIA* | *Rho GDP dissociation inhibitor (GDI) alpha; RHOGDI* | 17q25.3 | 18.6% | 18.6% | 113 |
| *AHCY* | *S-adenosylhomocysteine hydrolase* | 20cen-q13.1 | 9,0% | 9,0% | 21 |
| *TOP1* | *topoisomerase (DNA) I* | 20q12-q13 | 9,0% | 9,0% | 194 |
| *MYBL2* | *v-myb myeloblastosis viral oncogene homolog (avian)-like 2; BMYB* | 20q13.1 | 6.2% | 6.2% | 91 |
| *UBE2C* | *ubiquitin-conjugating enzyme E2C* | 20q13.11 | 6.2% | 6.2% | 129 |
|  |  |  |  |  |  |
|  |  |  |  |  |  |
| NOTE: # HUGO approved gene symbol and name | |  |  |  |  |
| * (Alers et al., 2001; Sattler et al., 1999; Steiner et al., 2002; Verdorfer et al., 2001; Wolter et al., 2002; Zitzelsberger et al., 2001) | | | | | |
| ** (Paris et al., 2003) | | |  |  |  |
| *** (Rhodes et al., 2002) | | | |  |  |
| § Ranking list of the 500 most up-regulated and down-regulated genes in prostate cancer vs. benign prostate tissue. | | | | | |
