# Supplementary material for: Expression analysis of imbalanced genes in prostate carcinoma using tissue microarrays
Source: Br J Cancer. 2006 Dec 5;96(1):82–8. doi: 10.1038/sj.bjc.6603490 (PMC2360197; doi:10.1038/sj.bjc.6603490)
Supplement: Supplementary Table 2.2 [file 6603490x2.doc]

| **Supplementary Table 2.2** Down-regulated genes (from top 250) on chromosomal bands with frequent (> 5%) losses | | | | | | |  |
| --- | --- | --- | --- | --- | --- | --- | --- |
|  |  |  | |  |  | |  |
|  |  |  | |  |  | |  |
|  |  |  | | **CGH*** | **Array CGH**** | | **Expression array meta-analysis***** |
|  |  | **Cromosomal** | | n=145 | n=16 | | n=61 |
| **Gene** # | **Gene Name** #**; Alias** | **location** | | **Losses** | **Losses** | | **Rank No.** § |
|  |  |  | |  |  | |  |
| *PAM* | *peptidylglycine alpha-amidating monooxygenase* | 5q14-q21 | | 9.0% | 1/16 | | 87 |
| *PJA2* | *praja 2, RING-H2 motif containing; KIAA0438* | 5q21.3 | | 9.0% | 2/16 | | 199 |
| *ITGA2* | *integrin alpha 2 (CD49B, alpha 2 subunit of VLA-2 receptor)* | 5q23-q31 | | 5.5% | 2/16 | | 152 |
| *FAM46A* | *family with sequence similarity 46, member A; FLJ20037* | 6q14 | | 13.1% | 2/16 | | 119 |
| *NT5E* | *5' nucleotidase, ecto (CD73)* | 6q14-q21 | | 17.2% | 6/16 | | 244 |
| *LAMA4* | *laminin, alpha 4* | 6q21 | | 15.2% | 2/16 | | 3 |
| *FYN* | *FYN oncogene related to SRC, FGR, YES* | 6q21 | | 15.2% | 2/16 | | 88 |
| *GJA1* | *gap junction protein alpha 1, 43kDa (connexin 43)* | 6q21-q23.2 | | 15.9% | 4/16 | | 9 |
| *PLN* | *phospholamban* | 6q22.1 | | 12.4% | 4/16 | | 223 |
| *AKAP12* | *A kinase (PRKA) anchor protein (gravin) 12* | 6q24-q25 | | 9.0% | 0/16 | | 39 |
| *PLAGL1* | *pleiomorphic adenoma gene-like 1* | 6q24-q25 | | 9.0% | 0/16 | | 53 |
| *TACC1* | *transforming acidic coiled-coil containing protein 1* | 8p11 | | 17.2% | 2/16 | | 13 |
| *RBPMS* | *RNA binding protein with multiple splicing* | 8p12-p11 | | 20.0% | 5/16 | | 86 |
| *PPP2CB* | *protein phosphatase 2 (formerly 2A), catalytic subunit, beta isoform* | 8p12-p11.2 | | 20.0% | 5/16 | | 67 |
| *CLU* | *clusterin (complement lysis inhibitor, SP-40,40, sulfated glycoprotein 2, testosterone-repressed prostate message 2, apolipoprotein J)* | 8p21-p12 | | 22.8% | 6/16 | | 200 |
| *DPYSL2* | *dihydropyrimidise-like 2* | 8p22-p21 | | 22.1% | 6/16 | | 98 |
| *GTF3A* | *general transcription factor IIIA* | 13q12.3-q13.1 | | 7.6% | 0/16 | | 162 |
| *SPG20* | *spastic paraplegia 20, spartin (Troyer syndrome); KIAA0610* | 13q13.1 | | 7.6% | 0/16 | | 205 |
| *AKAP11* | *A kinase (PRKA) anchor protein 11* | 13q13.3 | | 7.6% | 0/16 | | 239 |
| *FOXO1A* | *forkhead box O1A (rhabdomyosarcoma)* | 13q14.1 | | 12.4% | 3/16 | | 69 |
| *ESD* | *esterase D/formylglutathione hydrolase* | 13q14.1-q14.2 | | 12.4% | 3/16 | | 92 |
| *CYLD* | *cylindromatosis (turban tumour syndrome)* | 16q12.1 | | 9.7% | 0/16 | | 158 |
| *ARL2BP* | *ADP-ribosylation factor-like 2 binding protein; BART1* | 16q12.2 | | 9.7% | 0/16 | | 248 |
| *ALDOA* | *aldolase A fructose-bisphosphate* | 16q22-q24 | | 14.5% | 4/16 | | 222 |
| *FOXF1* | *forkhead box F1* | 16q24 | | 9.0% | 4/16 | | 157 |
| *SLC14A1* | *solute carrier family 14 (urea transporter) member 1 (Kidd blood group)* | 18q11-q12 | | 8.3% | 1/16 | | 124 |
| *MAPRE2* | *microtubule-associated protein, RP/EB family, member 2* | 18q12.1 | | 8.3% | 1/16 | | 212 |
| *ZNF516* | *zink finger protein 516; KIAA0222* | 18q23 | | 9.7% | 0/16 | | 207 |
|  |  |  | |  |  | |  |
|  |  |  | |  |  | |  |
| NOTE: # HUGO approved gene symbol | |  | |  |  | |  |
| * (Alers et al., 2001; Sattler et al., 1999; Steiner et al., 2002; Verdorfer et al., 2001; Wolter et al., 2002; Zitzelsberger et al., 2001) | | | | | | | |
| ** (Paris et al., 2003) | | |  | | |  |  |
| *** (Rhodes et al., 2002) | | | | | |  |  |
| § Ranking list of the 500 most up-regulated and down-regulated genes in prostate cancer vs. benign prostate tissue. | | | | | | | |
